# A conserved cysteine in the DNA-binding domain of MmuPV1 E2 is required for replication *in vivo*

**DOI:** 10.1128/jvi.01423-24

**Published:** 2024-12-12

**Authors:** Jessica Gonzalez, Kennedy Stoll, Marsha DeSmet, Elliot J. Androphy

**Affiliations:** 1 Department of Microbiology and Immunology, Indiana University School of Medicine 12250 , Indianapolis, Indiana, USA; 2 Indiana University School of Medicine 12250 , Terre Haute, Indiana, USA; 3 Department of Dermatology, Indiana University School of Medicine 12250 , Indianapolis, Indiana, USA; International Centre for Genetic Engineering and Biotechnology, Trieste, Italy

**Keywords:** papillomavirus, HPV, E2, DNA binding, transcription, replication, MmuPV1

## Abstract

The papillomavirus (PV) E2 protein is highly conserved, consisting of an N-terminal transactivation domain linked to a C-terminal DNA binding and dimerization domain (DBD) by a flexible hinge region. The E2 DBD exhibits a helix-turn-helix structure that dimerizes into a beta barrel prior to binding DNA; the first helix, α1, is responsible for recognition of the palindromic E2 binding site. The DNA recognition helix consists of a tract of basic amino acids with a highly conserved central cysteine residue. Previous mutational analysis studies on this conserved cysteine have found that it is not required for viral replication or DNA binding. To investigate the function of this conserved cysteine *in vitro* and *in vivo*, we generated point mutations in MmuPV1 E2 at cysteine 307. We report here that this cysteine in the DNA recognition helix is required for transient viral replication and transactivation of proximal promoters, but C307 point mutants are still capable of enhancing the activation of distant upstream promoters *in vitro*. MmuPV1 genomes with the C307 mutation failed to produce warts when injected into mice, suggesting that the DNA recognition cysteine is required for viral replication *in vivo*.

**IMPORTANCE:**

Papillomaviruses are the etiological agents of cancers of the oropharynx and anogenital tract. Understanding the mechanisms underlying PV pathogenesis is complicated by the strict species tropism displayed by the virus. The research presented here is significant because it links *in vitro* and *in vivo* models investigating the role of a conserved cysteine in the MmuPV1 E2 protein. This work elucidates the molecular mechanisms that regulate PV transcription and DNA replication and how these contribute to disease progression.

## INTRODUCTION

Papillomaviruses (PVs) are nonenveloped, double-stranded DNA viruses that infect the stratified epithelium of many vertebrate species. PVs tend to preferentially infect either the cutaneous or mucosal epithelium, though some can productively infect either niche (1). One such virus is the murine papillomavirus, MmuPV1. Isolated from the muzzle of athymic laboratory mice in 2010, the virus was initially believed to be cutaneous specific (2). However, further studies on the new virus revealed that MmuPV1 could infect either cutaneous or mucosal sites (3). Human papillomaviruses (HPVs) are categorized as low risk or high risk; infection with a low-risk HPV typically leads to development of benign epithelial lesions, which are spontaneously cleared by the host immune system. High-risk HPVs, on the other hand, have the potential to integrate into the host genome, a frequent first step toward HPV-mediated carcinogenesis (4). Persistent infection with a high-risk HPV is associated with cancers of the anogenital tract and the oropharynx (5). In addition to expanded tissue tropism, MmuPV1 is also capable of producing both benign and malignant infections (6, 7).

Four PV proteins are highly conserved: L1 and L2, the major and minor capsid proteins, respectively, and E1 and E2, both of which are required for replication of the viral genome (8). E2 is a multifunctional protein consisting of an N-terminal transactivation domain (TAD) and a C-terminal DNA binding and dimerization domain (DBD) connected by a flexible hinge region (9). The E2 DBD exhibits a helix-turn-helix structure; the first helix, α1, is responsible for recognition of the palindromic E2 binding site and consists of a tract of basic amino acids with a highly conserved central cysteine residue.

E2 dimers (10, 11) bind to the inverted palindromic sequence, ACC(N_6_)GGT (12), in which N represents any nucleotide. The E2 proteins from different genera tend to display differential preferences for the spacer sequence in the E2 binding site (13, 14); additionally, E2 has been shown to be capable of binding single-nucleotide variants of the conserved bases in the palindromic binding site (15). Several residues in the E2 DBD have been identified as necessary for specific recognition of the E2 binding motif, including the central cysteine residue, which forms hydrogen bonds with A(−6) and G(+5) of the E2 binding site (16). This cysteine within the DNA recognition helix is very highly conserved across papillomavirus types. Previous studies focusing on the DNA recognition cysteine in BPV-1 E2 have identified it as a site of redox activity, thereby regulating DNA binding of E2 (17). Additionally, it has been found that mutation of this cysteine in BPV-1 E2 abrogates transcriptional activation but not transient DNA replication, indicating a role for the DBD in regulating transcription (18). Here we present evidence that the DNA recognition cysteine in MmuPV1 E2 is required for promoter activation, transient replication, and efficient DNA binding *in vitro*, as well as replication and wart formation *in vivo*. However, low levels of binding to the MmuPV1 long control region (LCR) allow E2 cysteine mutants to activate distal promoters, suggesting that interaction with cellular factors at enhancer sites allows E2 to exert transcriptional control on the viral episome irrespective of DNA binding. Taken together, these findings suggest that modification of the DNA recognition cysteine in the E2 DBD may act as a regulatory switch for directing E2-mediated transcription and replication.

## RESULTS

### The DNA recognition cysteine is required for transcriptional activation

**Fig 1:**
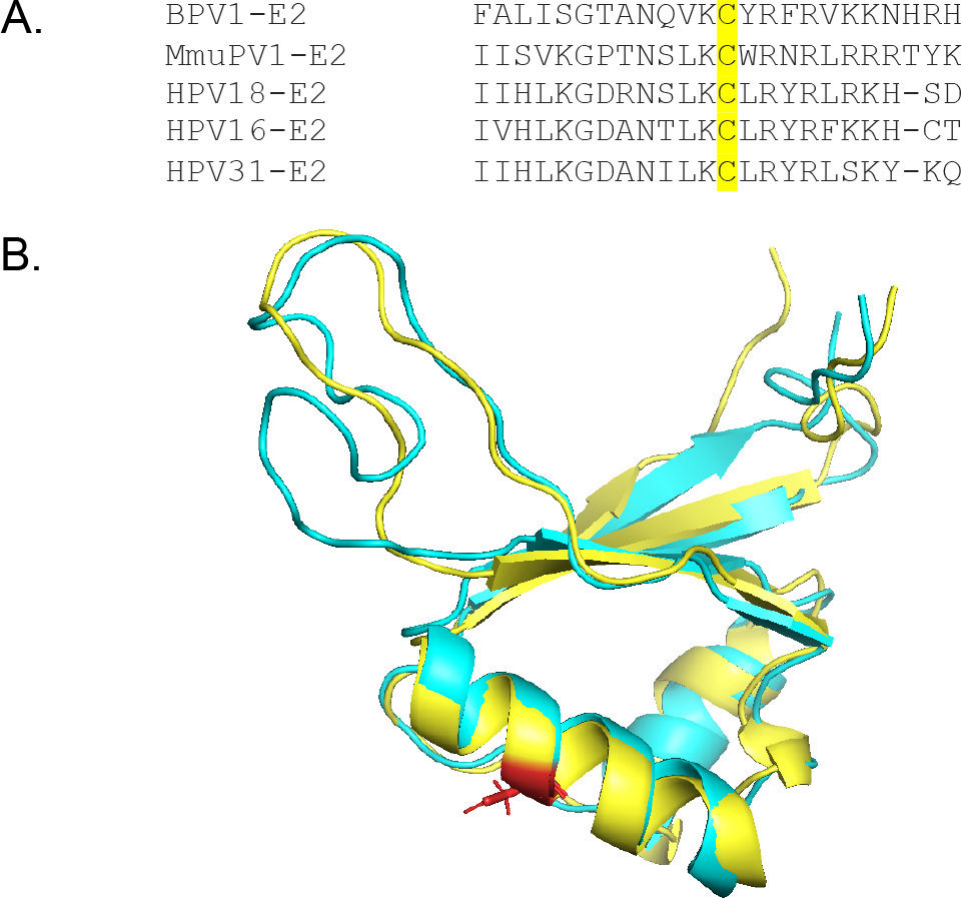
(**A**) The DNA recognition cysteine is conserved across multiple papillomavirus types. In MmuPV1, the cysteine is located at position 307. (**B**) Structure prediction of the MmuPV1 DBD was performed using I-TASSER-MTD. The MmuPV1 DBD (cyan) was aligned with the BPV-1 DBD (yellow) using PyMOL. The conserved cysteine is shown in red.

We first performed multiple sequence alignment to demonstrate that the central cysteine in the DNA recognition helix of E2 is conserved in MmuPV1 and across various papillomavirus types (Fig. 1A). Because the crystal structure has not been solved, we next utilized the multidomain structure prediction tool I-TASSER-MTD to predict the folding of MmuPV1 E2 (19). The predicted structure of the DBD was then aligned with the solved structure of the BPV-1 E2 DBD (PDB 1DBD); the overlaid structures were visualized in PyMOL, with MmuPV1 E2 in cyan and BPV-1 E2 in yellow, with the conserved cysteine in red (Fig. 1B) (20). The specific amino acids and their position within the DBD are necessary to mediate interaction with the E2 binding site (16). Because the cysteine is both conserved and located in the same structural context of the DBD, we hypothesized that it would similarly regulate MmuPV1 E2 function as has been observed in BPV-1 E2.

To investigate the role of the DNA recognition cysteine, we generated substitution mutants: C307S, the conservative mutation to serine that has been shown to be functional in BPV-1 E2, and C307F, a putative DNA binding-deficient phenylalanine mutant that would act as a negative control (18). We first investigated whether these mutants could activate transcription. Previous work on the homologous cysteine in BPV-1 E2 demonstrated that mutation to phenylalanine, serine, or glycine abrogated transcriptional activation functions when in the context of the full-length E2 protein. However, when the mutant DNA-binding domains were fused to the herpesvirus VP16 transactivation domain, the transcriptional activity of the serine and glycine mutants were restored. These results indicated the DNA recognition cysteine was not required for transactivation from a heterologous TAD (18).

**Fig 2:**
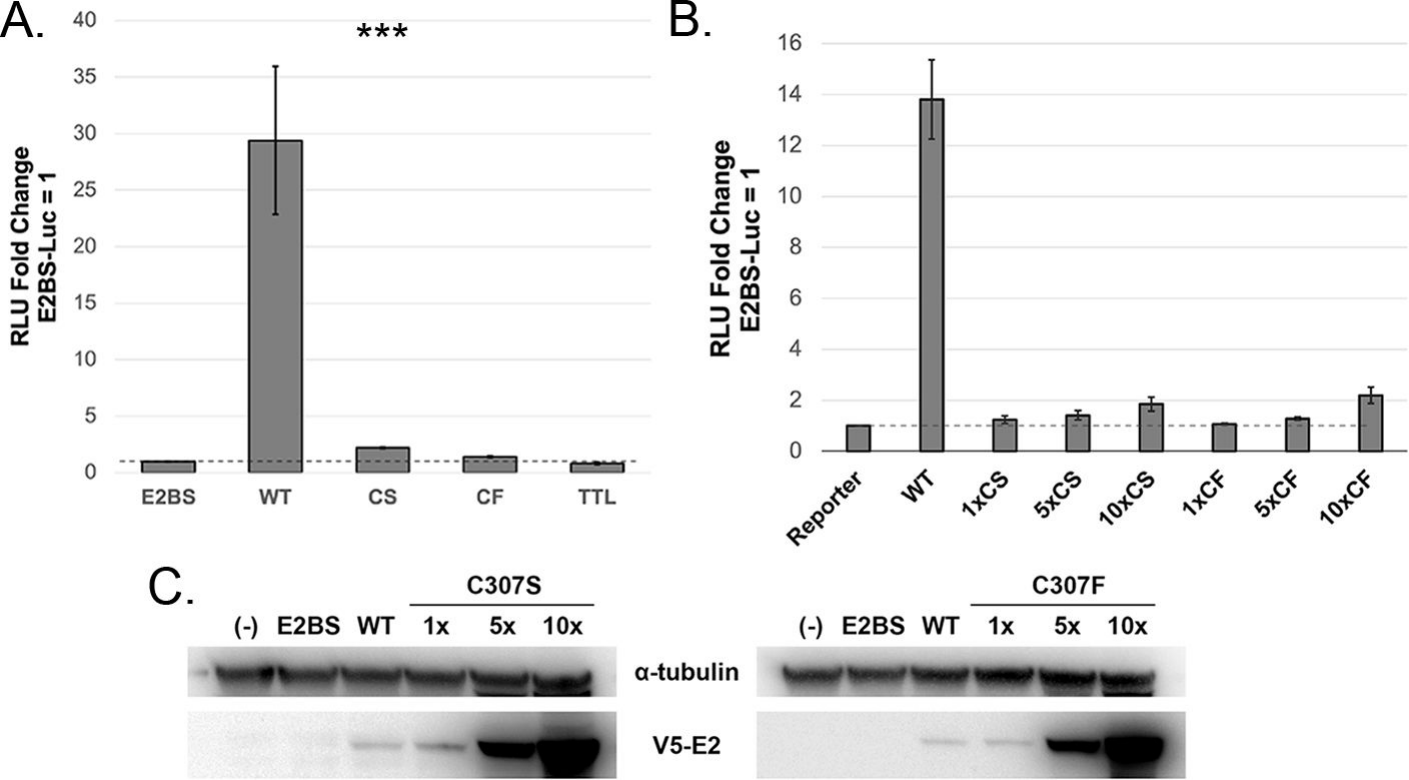
The DNA recognition cysteine is required for transcription activation. (**A**) Transcriptional activity of C307S and C307F mutants was assessed by luciferase assay. C33-A cells in a six-well plate were transfected with 200 ng of an E2-responsive luciferase reporter, pGL2-E2BS-Luc, and 100 ng either WT or mutant E2. Luciferase activity was measured 48 h post-transfection and was normalized to the reporter alone. (**B**) Transcriptional activity of the C307 mutants is not restored by overexpression. C33-A cells in a 12-well plate were transfected with 100-ng pGL2-E2BS-Luc and increasing amounts of the WT and mutant pCI-V5-E2 expression vectors (1×, 50 ng; 5×, 250 ng; and 10×, 500 ng). The empty pCI-V5 vector was used in the reporter only, 1× and 5× samples to maintain constant DNA concentrations across each transfection. (**C**) Whole-cell lysates from the overexpression assay were separated by SDS-PAGE. E2 was blotted with V5 antibody (Invitrogen #R960-25), and ⍺-tubulin was blotted as a loading control (DM1A). ***P < 0.001.

To assess the transactivation function of the C307 mutants, C33-A cells were transfected with a synthetic E2-responsive luciferase reporter, pGL-E2BS-Luc, and V5-tagged expression vectors for either wild-type (WT) or mutant E2 (21). An early termination mutant named translation termination linker (TTL) was included as an additional negative control. The C307S and C307F mutations in MmuPV1 E2 were incapable of activating transcription from this reporter compared to WT E2 (Fig. 2A; Table 1). The defect in transcriptional activity was not rescued by overexpression of the mutant E2 expression vectors, though both did reach significance slightly above background when overexpressed 10-fold (Fig. 2B).

**TABLE 1:** Summary of the replication and transcription activities of the MmuPV1 E2 C307 variants in C33-A cells^*a*^

	E1-dependent replication	Transcription			
		5′ E2BS	3′ E2BS	5′ LCR	3′ LCR
C307	+	+	+	+	+
C307S	−	−	Reduced	Reduced	+
C307F	−	−	−	−	Reduced

^*a*^ “+” indicates functionality; “−” indicates loss of function; “reduced” indicates a moderate defect compared to WT.

To ensure that the observed transcriptional defect was not due to decreased protein expression or instability of the mutants, we compared protein levels. The whole-cell lysate was separated by SDS-PAGE and immunoblotted with antibodies to V5 and alpha tubulin as a loading control (Fig. 2C). Both C307 mutants were expressed at levels comparable to WT E2 at 1× vector concentration, and both displayed dose-dependent increases in protein expression, indicating that the mutations did not negatively affect protein expression or stability. From this, we concluded that E2 C307S and C307F are severely deficient for transactivation function, which cannot be compensated for by overexpression of each protein.

### C307 mutants localize to the nucleus

**Fig 3:**
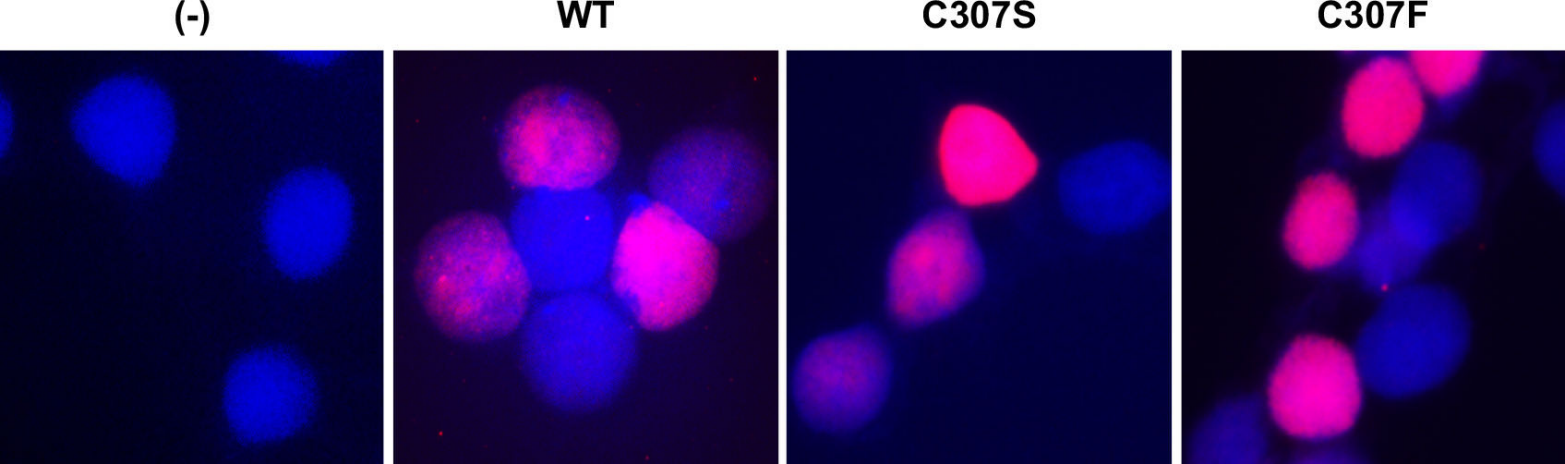
C307 is not required for nuclear localization of E2. HEK293TT cells were transfected with 3 µg of either WT, C307S, or C307F pCI-V5-E2 expression vectors. Cells were fixed approximately 48 h post-transfection. Cells were stained for V5 (CST D3H8Q) and mounted with DAPI. Slides were analyzed for localization of E2 (red) within the nucleus (blue).

The E2 protein of various PVs is localized to the nucleus (22, 23). A previous study of BPV-1 E2 has established that one of the protein’s nuclear localization signals may be in the DNA recognition helix of the DNA-binding domain (24). Additionally, in studies on BPV-1 E2, the cysteine point mutants were all localized to both the nucleus and the cytoplasm, whereas the WT E2 was primarily nuclear (18). To ensure that mutation of C307 did not interfere with protein localization, immunofluorescence was performed (Fig. 3). HEK293TT cells were plated on glass coverslips and transfected with WT or C307 mutant E2 expression vectors. Cells were fixed and stained with V5 antibody approximately 48 h post-transfection. Primary antibody was removed and cells were washed in phosphate-buffered saline (PBS) prior to incubation with AlexaFluor 555 anti-Rb (#A21428) for 2 h. Secondary antibody was removed and cells were washed 3× in PBS prior to being mounted with ProLong Gold containing 4′,6-diamidino-2-phenylindole (DAPI). Both E2 C307S and C307F proteins localized primarily to the nucleus, demonstrating that the defect in transactivation is not explained by mislocalization.

### C307 is required for transient DNA replication

E2 is one of two viral proteins required for viral genome replication (8). The other protein, E1, is the viral helicase necessary for unwinding of the viral origin (25). When expressed together, E2 can bind and recruit E1 to the viral origin of replication, whereupon the replication complex can form (26). We next investigated whether mutation of C307 would affect its transient DNA replication functions. We hypothesized that C307S would be capable of supporting transient replication, as has been demonstrated previously in BPV-1 E2 (18). We utilized the luciferase-based transient replication assay developed by the Archambault group (27). We generated an E2-responsive reporter called mFLori for MmuPV1 firefly luciferase origin, which contains the complete MmuPV1 LCR downstream of the firefly luciferase gene. Because the LCR contains the origin of replication, when mFLori is co-transfected with E2 and E1 the replication complex can assemble and replicate the mFLori reporter. The reporter pRLuc, which encodes the Renilla luciferase, is a control for transfection efficiency. The ratio of firefly luciferase to Renilla luciferase is a measure for E1- and E2-mediated replication.

**Fig 4:**
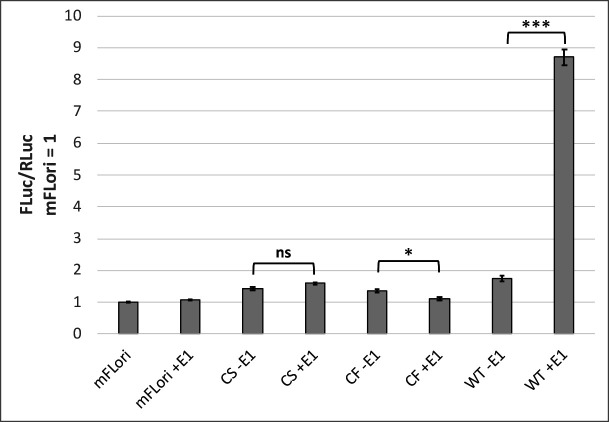
The DNA recognition cysteine is necessary for transient replication. C33-A cells in a 96-well plate were transfected with mFLori (5.6 ng) and pRLuc (1.4 ng) and either C307S, C307F, or WT pCI-V5-E2 (14 ng). Each E2 variant was tested with an E1 early termination mutant (labeled –E1) or with pCI-myc-E1 (28 ng). Values are reported as the mean fold change across three separate assays. Error bars represent the SEM. Pairwise *t*-tests were performed between the matched E2 samples with or without E1. *P < 0.05, ***P < 0.001.

C33-A cells were transfected with WT or C307 mutant E2 expression vectors, mFLori, pRLuc, and either WT or TTL E1 expression vectors. The ratios of FLuc:RLuc were compared between samples containing either TTL or WT E1; the samples containing TTL E1 represent the background transactivation activity that can be attributed to E2 alone. All samples were normalized to cells containing only mFLori and pRLuc to account for background luciferase activity from the reporters. The FLuc:RLuc ratio of WT E2 expressed alongside WT E1 was increased approximately eightfold relative to the reporters alone and was significantly increased compared to samples containing WT E2 and TTL E1, indicating successful transient DNA replication. Conversely, neither C307S nor C307F had FLuc:RLuc ratios that were significantly increased when compared with the matched TTL E1 samples, indicating that neither C307S nor C307F were able to induce transient replication (Fig. 4; Table 1). Interestingly, previous study on the BPV-1 E2 DBD found that mutation of the homologous cysteine did not abrogate transient DNA replication, while substitution to phenylalanine decreased replication activity (18). This suggests that the DNA recognition cysteine may have distinct functions in differing PV types.

### C307 mutants activate transcription from the mFLori reporter

**Fig 5:**
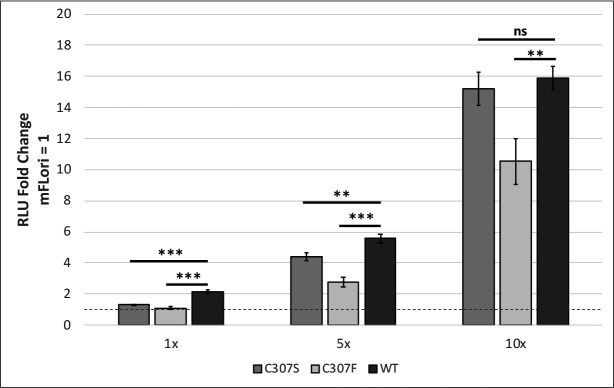
C307 mutants can activate transcription from the mFLori reporter. C33-A cells in a 12-well plate were transfected with increasing amounts of either WT, C307S, or C307F pCI-V5-E2 (1×, 50 ng; 5×, 250 ng; and 10×, 250 ng) and the E2 binding site reporter mFLori (20 ng). Cells were lysed approximately 48 h post-transfection, and transactivation was measured by firefly luciferase assay. Fold change was calculated relative to cells containing the reporter alone. Values are reported as the mean fold change across three separate assays. Error bars represent the SEM.**P < 0.01, ***P < 0.001.

During our experiments to investigate transient DNA replication, it was noted that addition of E1 reduced the FLuc:RLuc ratio in C307F, implying that an excess of E1 leads to a decrease in background transactivation from E2 alone (Fig. 4). Because we had found that the C307 mutants were incapable of activating transcription even at 10-fold overexpression, we were surprised to detect any background in this low E2 system. We next repeated the transcriptional activation assay, this time using mFLori as the Luciferase reporter in place of pGL2-E2BS-Luc. Surprisingly, both C307 mutants activated transcription from the mFLori reporter in a dose-dependent manner (Fig. 5). Both mutants were inactive at the 1× and 5× expression levels, but at the 10× expression level, C307S was comparable to the transcriptional activity seen from WT E2. C307F also displayed statistically significant transcription at the 10× expression level, though it was unable to reach levels comparable to WT activity.

### C307 mutants show reduced DNA binding

**Fig 6:**
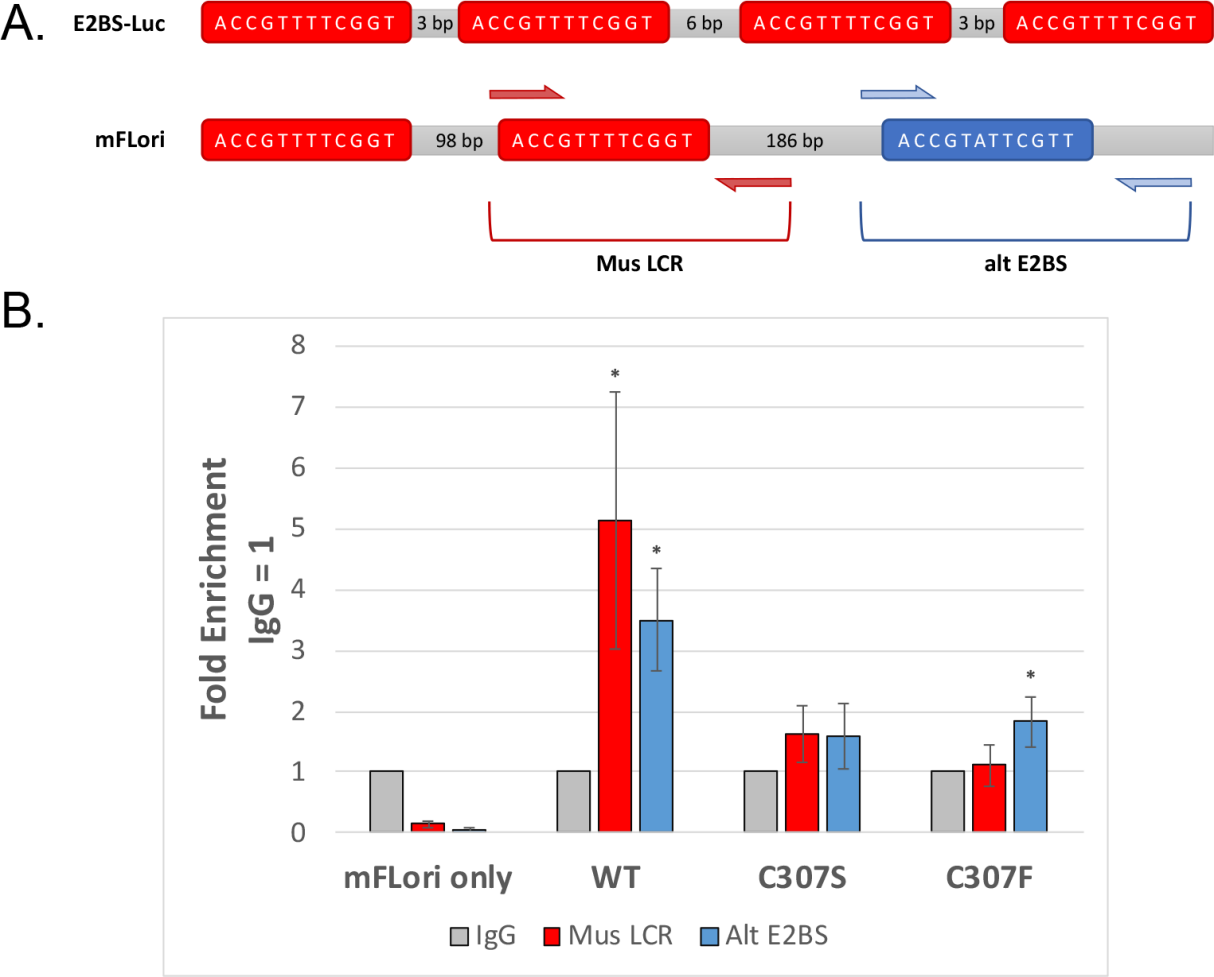
C307 mutants show reduced DNA binding. (**A**) Diagram of the E2 binding site layout found in pGL2-E2BS-Luc (top) and in mFLori (bottom). Approximate location of primer sets used for chromatin immunoprecipitation is indicated on the mFLori diagram. (**B**) Chromatin immunoprecipitation of WT, C307S, and C307F V5-E2. C33-A cells in 7-cm plates were transfected with mFLori (100 ng) and WT or mutant V5-E2 (7 µg). Immunoprecipitation was performed with antibodies against V5 or IgG as the control. The amount of E2 at each binding site was determined using primers flanking either site. Binding is represented as fold enrichment relative to IgG. Data are presented as the average fold enrichment across three independent replicates, and error bars represent the SEM. *P < 0.05.

Due to the stark contrast between transcription of the E2BS-Luc and mFLori reporters, we next sought to investigate why mFLori, but not E2BS-Luc, could act as a transactivation target for the C307 mutants. When comparing the two reporters, we noted that mFLori has an “alternative” binding site; this E2 binding site, BS3, is an imperfect palindrome and overlaps the promoter for MmuPV1 E6 (Fig. 6A). We hypothesized that the DNA recognition cysteine may play a role in binding site selection; the imperfect palindrome found in mFLori may allow for more flexibility in E2 binding, thereby accommodating the C307 mutants.

To investigate the DNA-binding capability and binding site preference of the MmuPV1 C307 mutants in live cells, we performed chromatin immunoprecipitation. C33-A cells were transfected with WT or mutant E2 expression vectors, in addition to mFLori, to act as an E2 binding target. Approximately 48 h post-transfection, cells were collected and immunoprecipitated using either V5 antibody or mouse IgG as a nonspecific control. Real-time quantitative PCR was performed using primers flanking one of the palindromic binding sites, BS2, or the alternative binding site, BS3. WT E2 was highly enriched at both binding sites, while both mutants displayed greatly reduced DNA binding at both sites. C307S was bound at low levels on both binding sites, but this was not significant. C307F did display a slight enrichment on BS3 compared to BS2, which was nearly equivalent to the IgG control. The binding of C307F on BS3 was statistically significant, which may indicate a moderate binding preference for the alternative binding site.

### C307 mutants activate transcription when the MmuPV1 LCR is downstream but not upstream of the luciferase promoter

**Fig 7:**
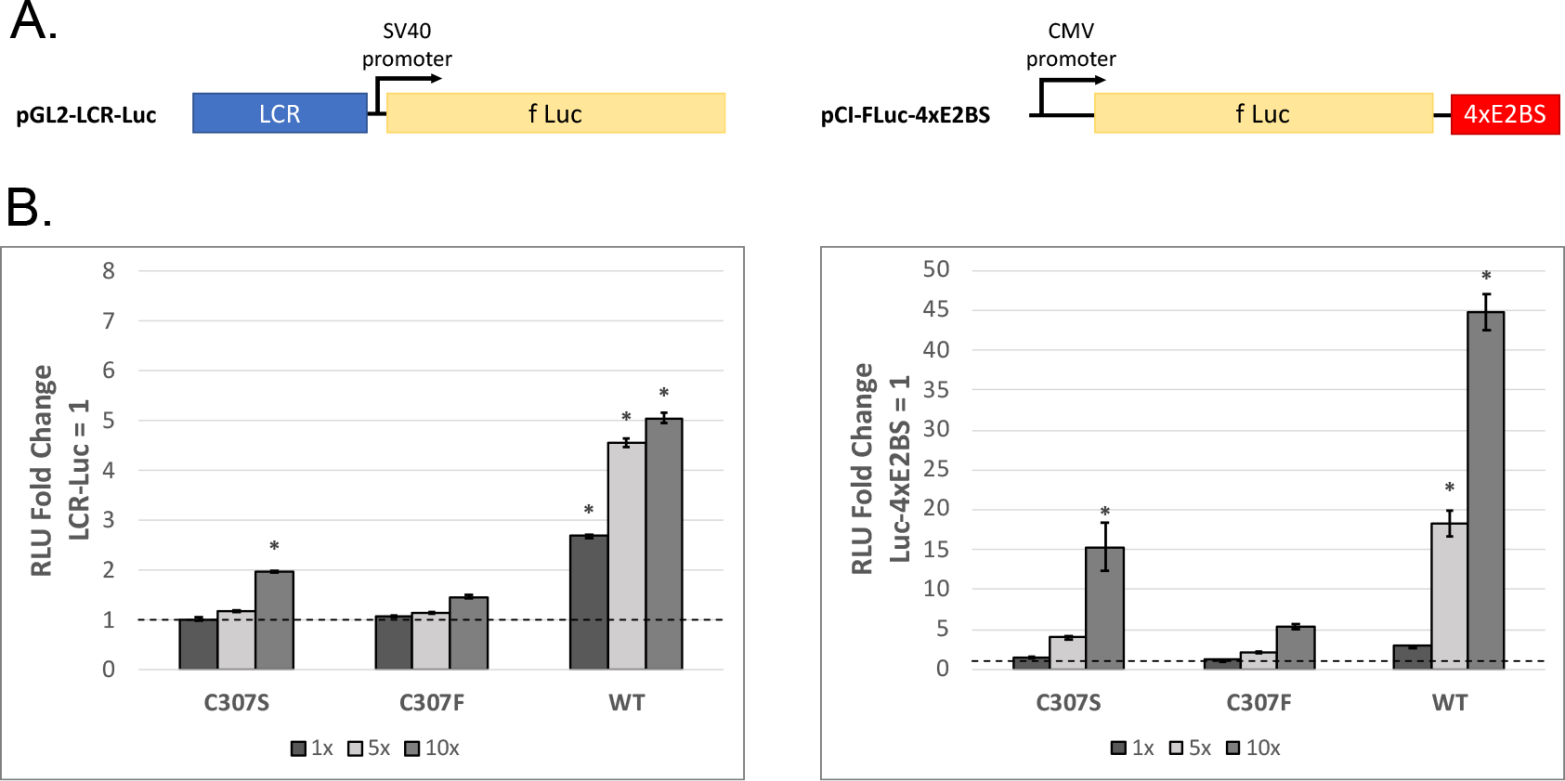
C307 mutants are defective for activating transcription from alternative luciferase reporters. (**A**) Diagram of the newly constructed E2 binding site reporters pGL2-LCR-Luc (left) and pCI-Luc-4xE2BS (right), indicating the approximate location of the E2 binding region relative to firefly luciferase. (**B**) Luciferase-based transcription assays were performed on C33-A cells in 12-well plates using increasing concentrations of C307S, C307F, and WT E2 (1×, 50 ng; 5×, 250 ng; and 10×, 500 ng) with each of the two reporters (pGL2-LCR-Luc, 150 ng, and pCI-fLuc-4xE2BS, 10 ng). Fold change was calculated relative to cells containing the reporter alone. Values are reported as the mean fold change across three separate assays. Error bars represent the SEM. Significance was determined by analysis of variance followed by Tukey’s honestly significant difference test. *P < 0.05.

Another major difference between the pGL2-E2BS-Luc and mFLori reporters is the location of the E2 binding sites; in E2BS-Luc there are four closely spaced E2 binding sites upstream of the luciferase promoter, while in mFLori the MmuPV1 LCR, containing three E2 binding sites, is downstream of luciferase. To investigate whether the position of the E2 binding sites in the reporter makes a difference in C307 mutant activity, we generated the reporters pGL2-LCR-Luc, which contains the MmuPV1 LCR upstream of the luciferase promoter, and pCI-FLuc-4xE2BS, which contains the four high-affinity E2 binding sites downstream of luciferase (Fig. 7A). We hypothesized that if the activity observed from the mFLori reporter was due to more permissive binding on the LCR, then we would see a similar pattern of activity when the LCR was upstream of luciferase.

We repeated the transactivation assay using these new reporters and overexpression of WT or mutant E2. Interestingly, the results from these assays more closely resembled those seen from the pGL2-E2BS-Luc reporter. WT E2 demonstrated significant dose-dependent transactivation, while C307S and C307F were both severely deficient for transcription, though C307S activity did reach significance at 10× overexpression (Fig. 7B). From this we concluded that the C307 mutants can transactivate from the LCR specifically when it is downstream but not immediately upstream of the luciferase promoter. Because the activity on mFLori did not correspond with strong binding of the C307 mutants, we conclude that the transcriptional enhancement is not strictly dependent upon high-affinity E2 DNA binding.

### The transcriptional activity of C307 mutants is cell type specific

**Fig 8:**
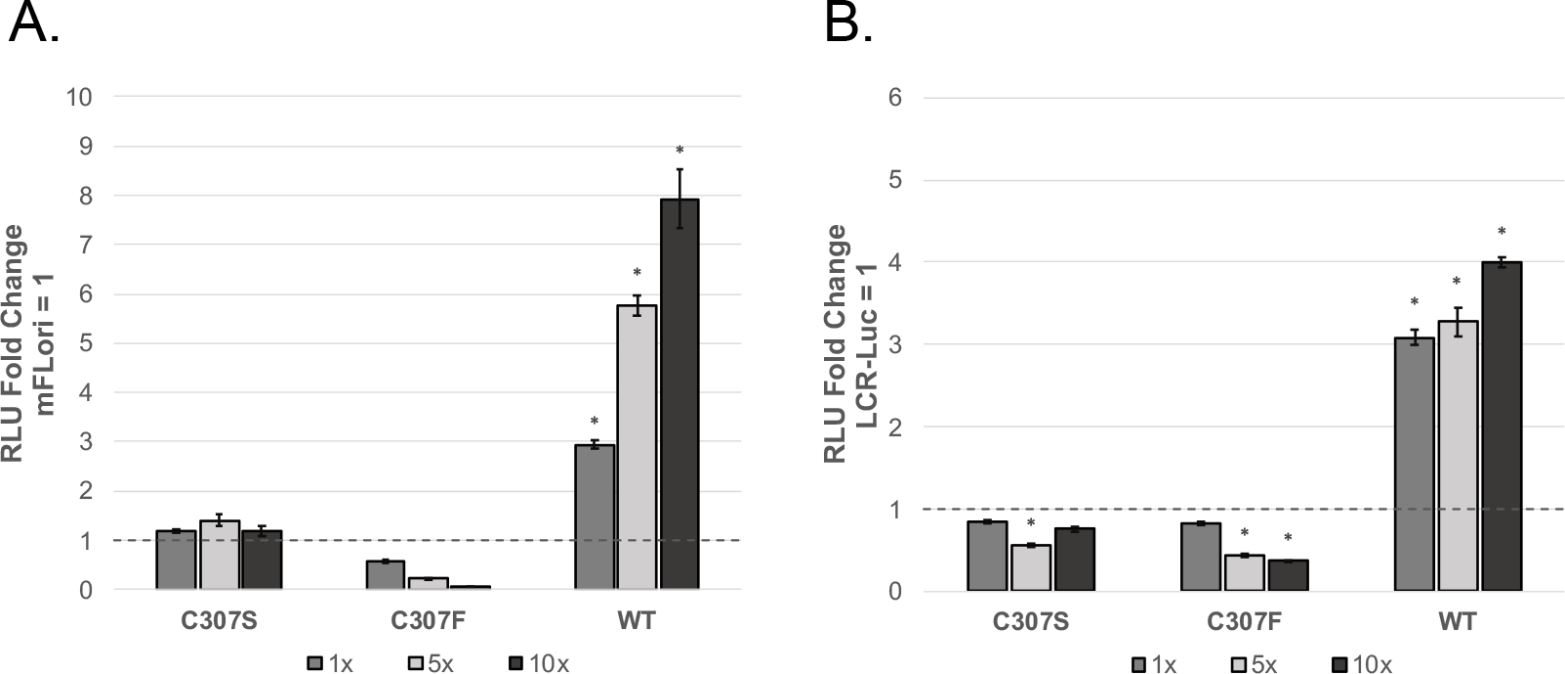
Transcriptional activity of the C307 mutants is cell type specific. 3T3-J2 cells in a 12-well plate were transfected and assessed by luciferase assay as described above. WT and C307 mutant V5-E2 were overexpressed in the presence of the mFLori (**A**) and pGL2-LCR-Luc (**B**) reporters to determine transcriptional activity. Fold change was calculated relative to samples containing only the reporter. Values are reported as the mean fold change across three independent assays, and error bars represent the SEM. Significance was determined by analysis of variance followed by Tukey’s HSD test. *P < 0.05.

We next assessed the transcriptional activity of the C307 mutants in the murine fibroblast cell line NIH 3T3-J2. Unlike C33-A cells, 3T3-J2 cells express easily detectable levels of Brm/Smarca2 (28). We chose to focus on the mFLori and pGL2-LCR-Luc reporters due to the presence of the full-length MmuPV1 LCR. In 3T3-J2 cells, WT V5-E2 activated transcription in a dose-dependent manner when overexpressed in the presence of either promoter. Neither mutant was able to activate transcription of the mFLori reporter (Fig. 8A) or the pGL2-LCR-Luc reporter (Fig. 8B). The results from C307F samples suggest that this mutant may repress transcription in a dose-dependent manner, though this was statistically significant only in assays with pGL2-LCR-Luc and not those with the mFLori reporter.

### C307 mutants are incapable of inducing wart formation

**Fig 9:**
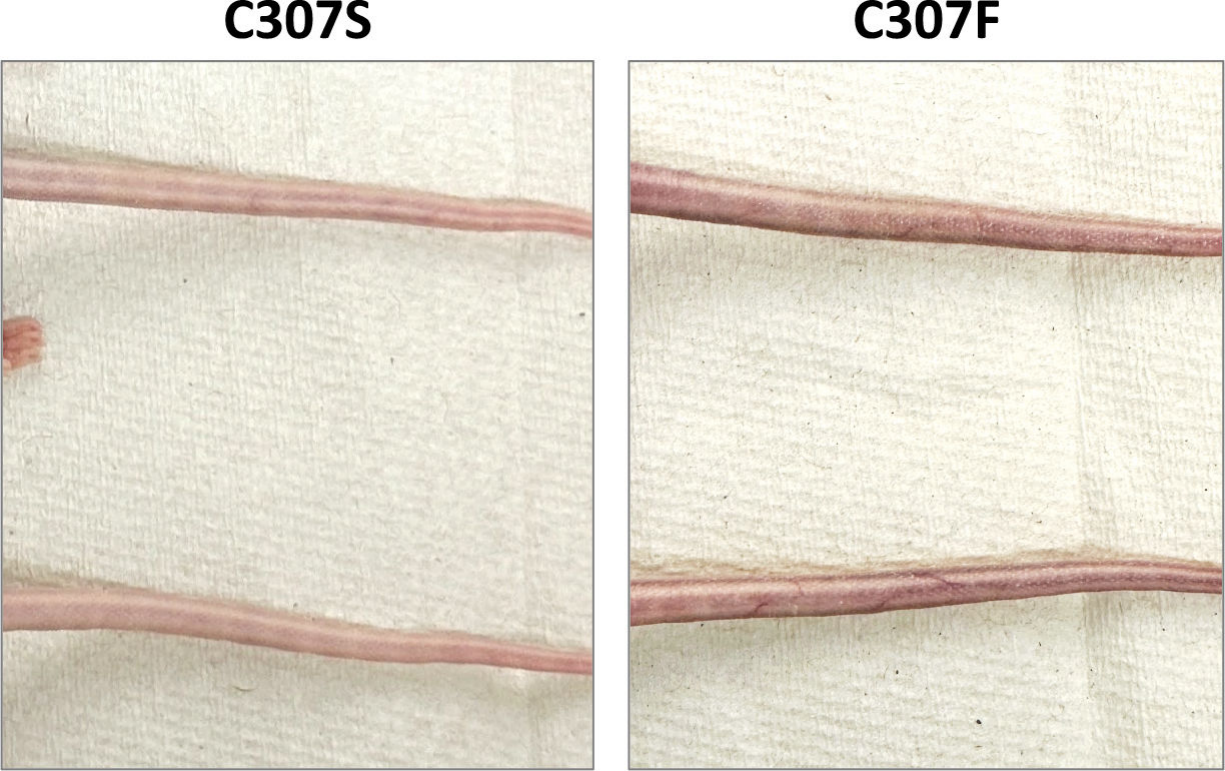
C307 mutants are unable to induce wart development *in vivo*. Images were taken at the conclusion of the experiment, approximately 5.5 months post-injection. Neither mutant produced verrucous lesions.

Because the mFLori reporter contains the native LCR of MmuPV1 and both E2 mutants displayed some transcriptional activity from this reporter, we questioned whether cysteine 307 is necessary to establish infection *in vivo*. To test this, we injected the tails of athymic hairless mice intradermally with approximately 18 µg of recircularized genomes containing the C307 mutant E2. Circularized genomes were prepared as previously described (29). Time-matched WT injections were excluded to minimize the risk of cross-contamination and viral spread within the animal facility. Mice were monitored weekly to assess disease progression. Our experiments utilizing the wild-type MmuPV1 genome demonstrated that mice develop cutaneous lesions anytime from 3.5 to 5.5 months post-injection (data not shown). In contrast to other E2 mutations in the context of the MmuPV1 genome (J. Gonzalez, M. DeSmet, and E. J. Androphy, unpublished data) that cause proliferative warts, after 5.5 months, neither C307 mutant produced cutaneous lesions at the injection site nor at distal anatomic sites (Fig. 9). Because PVs can cause asymptomatic infections, DNA was isolated from the epidermis of the tail using the Qiagen DNeasy Blood and Tissue Kit. The DNA was then used as template in PCR with primers to amplify regions in E1 and E2. Neither C307 mutant yielded viral DNA in the tissue samples, indicating that neither mutant genome was able to establish or maintain an asymptomatic infection. We conclude that the E2 DBD cysteine is required for wart formation *in vivo*, and neither mutant can maintain an asymptomatic infection.

## DISCUSSION

The papillomavirus E2 protein is a multifunctional protein that functions in transcription, replication, and genome segregation. E2 consists of two functional domains—the N-terminal TAD and the C-terminal DBD—connected by a flexible hinge region. The E2 DBD consists of a helix-turn-helix motif; the first helix, α1, is the DNA recognition helix and is responsible for recognizing and binding the consensus E2 binding site. The amino acid sequence of the DNA recognition helix consists of a tract of basic residues with a central cysteine residue. This amino acid motif is highly conserved in the E2 DBD across many papillomavirus types and is also present in the DBD of cellular transcription factors, including ATF-1 and c-Jun (30, 31).

Serine (S) and phenylalanine (F) mutations were generated at C307 in MmuPV1 E2 to investigate the role of this highly conserved cysteine. *In vitro* studies showed that C307 is required for E1-dependent viral replication and E2-dependent transactivation of the pGL2-E2BS-Luc reporter, which contains four closely spaced E2 binding sites upstream of the promoter for firefly luciferase. Further experiments revealed that both C307 mutants could activate transcription from the downstream MmuPV1 LCR when overexpressed, but only C307S displayed transcriptional activity when the LCR was positioned upstream of the luciferase promoter. The results of the *in vitro* transient replication and transactivation assays conducted in C33-A cells are summarized in Table 1. Previous mutagenic studies of the homologous cysteine in BPV-1 E2 found that it was required for transcriptional activation but not replication (18). In contrast, the studies presented here suggest that C307 in MmuPV1 E2 is absolutely necessary for replication both *in vitro* (Fig. 4) and *in vivo* (Fig. 9).

The crystal structure of BPV-1 E2 in complex with DNA has shown that the DNA recognition cysteine is one of the four amino acids that makes a direct, discriminating contact with bases in the E2 binding site (16). One of these bases is G(+5), which is one of the bases that is altered in BS3 of the MmuPV1 LCR. Additionally, a previous report found that the homologous DNA recognition cysteine in BPV-1 E2 confers redox sensitivity to DNA binding; mutation of cysteine to serine, alanine, or glycine abrogated redox sensitivity, though all mutants displayed decreased DNA-binding affinity (17). As infected keratinocytes transit through the layers of the epithelium, they are exposed to an increasingly oxidizing environment (32), which alters cellular gene expression by regulating transcription factor activity (33, 34). Taken together, this suggested that E2 binding to DNA may be similarly altered by the oxidizing host cell, thereby regulating E2 activity based on the stage of the viral life cycle. This led to the hypothesis that MmuPV1 E2 C307 is involved in binding site selection. Chromatin immunoprecipitation assays (Fig. 6) showed that the WT E2 could significantly bind both the perfect and imperfect palindrome binding sites; C307S bound both sites, but this was not statistically significant. C307F displayed a slight binding preference for the imperfect palindrome. The reduced DNA binding of the C307 mutants could explain why these were only functional when overexpressed: as the mutant protein concentration increases, this could drive equilibrium toward increased binding site occupancy, thereby compensating for decreased binding affinity. While this would explain the ability of the C307 mutants to transactivate the mFLori reporter when overexpressed, this does not account for the inability of C307S to activate the pGL2-E2BS-Luc reporter when overexpressed.

E2 can activate transcription from downstream enhancers. In addition, the viral LCR has been shown to act as an E2-responsive enhancer independent of both position and orientation to a promoter (35, 36). The PV LCR contains binding sites for various cellular transcription factors, creating E2-independent, cell type-specific enhancers known as epithelial specific enhancers, which may contribute to the strict cellular tropism displayed by many PVs (37). The mFLori reporter was constructed by cloning the entirety of the MmuPV1 LCR downstream of firefly luciferase; in contrast, the HPV-31 replication reporter pFLORI31 contains only the portion of the LCR containing the viral origin of replication (27). The inclusion of the full-length MmuPV1 LCR in mFLori likely accounts for the high levels of E2-mediated transcription activation in C33-A-based assays with this reporter. Interestingly, when the LCR-containing reporters were assessed in the murine fibroblast cell line 3T3-J2, neither C307 mutant induced transcription of mFLori (Fig. 8A) or pGL2-LCR-Luc (Fig. 8B). Notably, C307F instead appeared to repress transcription in a dose-dependent manner. This may occur by “squelching” where E2 binds to a cellular transcription factor but is unable to localize this to the viral promoter (38). By contrast, in the epithelial cell line C33-A, both mutants were able to activate transcription of mFLori in a dose-dependent manner (Fig. 5). Analysis of the full-length HPV-18 LCR has suggested that it may contain regulatory elements that repress E2-inducible enhancers in some cell types but not in others (39). Thus, the differences we observed between C33-A and 3T3-J2 cells can likely be attributed to cell-type specificity of the enhancer elements within the MmuPV1 LCR.

Manipulation of genome architecture is one strategy by which a number of viruses ensure their persistence within the host cell (40). The Epstein-Barr virus episome is known to assume different genome conformations in the various forms of viral latency, thereby regulating gene expression (41). Furthermore, the EBNA3A and EBNA3C proteins have been proven to mediate long-range chromatin interactions of the host genome at various loci (reviewed in reference 42). Interestingly, the EBNA3 proteins regulate gene expression without direct DNA binding but rather through interactions with host transcription factors.

The latency-associated nuclear antigen (LANA) protein of Kaposi’s sarcoma-associated herpesvirus (KSHV) is capable of modulating transcription either through interaction with cellular transcription factors or direct promoter binding (43). A recent study investigating the genome-wide architecture in primary effusion lymphoma cells harboring KSHV found that LANA plays a role in intrachromosomal looping and regulation of enhancer-promoter interactions (44). This study identified LANA-mediating chromatin interactions between distant regions of the viral genome as well as within the host genome.

Multiple PV types have demonstrated E2-mediated DNA looping as a regulatory mechanism. E2 dimers from BPV-1 (45), HPV-16 (46), and HPV-11 (47) can mediate the formation of DNA loops between distant E2 binding sites. The HPV-31 genome has also been shown to undergo chromatin remodeling upon differentiation of the host cell that allows transcriptional machinery to access the late promoter (48). Furthermore, BPV-1 E2 interacts with the hBrm-containing SWI/SNF chromatin remodeling complex to enhance transcriptional activation (21). SWI/SNF chromatin remodeling functions by altering the position of nucleosomes on DNA, thereby making genes accessible for expression. While it is possible that MmuPV1 E2 also interacts with Brm, the majority of the experiments presented in our work were conducted in C33-A cells, which are SWI/SNF defective and do not express Brm to detectable levels (49). Thus, the mechanism of E2-mediated enhancement presented here is novel.

We propose that elements within the MmuPV1 LCR allow E2 C307 mutants to activate upstream, but not downstream, promoters. The activation appears to be independent of binding affinity, as demonstrated by C307S transactivation matching WT activity on mFLori despite low binding. C307F demonstrated activity on mFLori when overexpressed despite very low DNA binding. This suggests that post-translational modifications on this cysteine can act as a way to regulate, but not wholly eliminate, E2 transcriptional activity on distant promoters. Together, these data suggest that modifications of C307 may be one mechanism by which the virus controls gene expression throughout its life cycle.

While the evidence suggests E2-mediated enhancement, we have not investigated the chromosome architecture to confirm this finding. Chromatin conformation capture experiments might elucidate an interaction between the LCR and luciferase promoter that is enhanced by E2 expression. Previous studies on the conserved cysteine in the E2 DNA recognition helix were conducted using BPV-1 E2 and primarily utilized fusion proteins or protein fragments. Here, we present insight into the function of the DNA recognition cysteine in MmuPV1 E2 in the context of the full-length protein. In addition, this is the first study investigating the E2 DBD cysteine using an *in vivo* model. By studying C307 using both *in vitro* and *in vivo* techniques, we can conclude that the DNA recognition cysteine in MmuPV1 E2 is required for replication and proliferative wart formation. Future studies should be conducted to investigate the function of the DNA recognition cysteine in other papillomavirus types; this cysteine is conserved in high-risk HPVs, including HPV-16, HPV-18, and HPV-31, and study of the DNA recognition cysteine in these viruses could yield insight into how E2 DNA binding and transactivation are regulated in high-risk HPVs.

## MATERIALS AND METHODS

### Plasmids and mutagenesis

The pMusPV plasmid was provided by John Schiller. The pGL2-4xE2BS-Luc (21) and pCI-Fluc (pFLuc) (27) have been described previously. The pUC19-E2 plasmid was generated by digesting pMusPV with KpnI and cloning into the KpnI site in pUC19. Site-directed mutagenesis was performed on pUC19-E2 using the NEB Q5 Site-Directed Mutagenesis Kit (NEB #M0492S) with the mutagenic primers C307S Fwd: CTCTTTAAAATCCTGGCGGAATAG, C307F Fwd: CTCTTTAAAATTCTGGCGGAATAG, and C307 Rev: TTAGTCGGACCTTTGACAG. TTL E2 was generated by site-directed mutagenesis using primers E2 TTL Fwd: CGATGCAGTGTAAGACCAGATAC and E2 TTL Rev: AAACGTGTTTCCAGGCTG.

WT and mutant E2 ORFs were PCR amplified from pUC19-E2 using primers V5-E2 Fwd: ATGCGAATTCATGAACAGCCTGGAAACACGTTT and V5-E2 Rev: ATGCGTCGACTCAGAGTCCGTCTAAGAAGC. PCR products and the pCI-V5 vector were digested with EcoRI and SalI prior to ligation to generate the V5-E2 expression vectors. To generate mutant viral genomes, the C307 mutations were amplified from pUC19-E2 using primers E2-L2 Fwd: ACAAACCTGCATCCGGAAAG and E2-L2 Rev: ATCGGGGGTCACCTCAAG. PCR products and the pMusPV1 plasmid were digested with MroI and Eco91I prior to ligation. All genomes were sequenced in full to ensure no off-target mutations were generated.

The mFLori plasmid was generated by amplifying the MmuPV1 LCR from pMusPV using primers Mus ori Fwd: ATGCGCCGGCCTGAACTGGTGCTACTAACTGAATGAC and Mus ori Rev: ATGCGCCGGCGAACGAATACGGTTATGGGGG. The PCR product was digested and ligated into the NgoMIV site in pFLuc. The reporter pGL2-LCR-Luc was generated by amplifying the MmuPV1 LCR from mFLori using primers Fwd: GTCACTGCAGCTGAACTGGTGCTACTAACTG and Rev: ACGTCGGACCGGGCGAACGAATACGG. The PCR product was digested with SbfI and CspI and ligated into the PstI and CspI sites in pGL2-E2BS-Luc.

The pCI-FLuc-4xE2BS reporter was generated by amplifying the four E2 binding sites from pGL2-E2BS-Luc with primers Fwd: ACGTGCCGGCGCGCAGGTCTACAACCGTTTTC and Rev: ACGTGCCGGCCTAGAGTCGGACCGAAAAC. The PCR product was digested and ligated into the NgoMIV site in pFLuc.

To generate the early termination E1 mutant, the partial open reading frame of E1 was amplified from pMusPV1 using primers Fwd: ATCGGTCGACTCTCGAGGAGGTGCTTAG and Rev: ACGTGTCGACGCGTTAGATCGGTGAAG. The PCR product was digested and cloned into the SalI site of pUC19 to generate pUC-partial-E1. Site-directed mutagenesis was then performed on pUC-partial-E1 using the primers Fwd: CAGGGCAGTAATCTGGATGGT and Rev: TACCTTTATCGTTTTCCATTTCTG. The mutated E1 fragment was then ligated back into the MluI and XhoI sites of pMusPV1 to generate the TTL E1 mutant pMusPV1 genome. The E1 open reading frame was amplified from either the WT or TTL mutant pMusPV1 genome using the primers E1 Fwd: ACGTGAATTCATGGAAAACGATAAATGTACAGGGCAG and E1 Rev: ACGTGTCGACTTACTGCCTTTCTCGTAAAGGTTC. Fragments were digested and subsequently cloned into the EcoRI and SalI sites in pCI-myc to generate the expression vectors pCI-myc-E1 and pCI-myc-E1 TTL.

### Cell culture and transfection

All cell lines were maintained at 37°C and 5% CO_2_. HEK293TT, NIH 3T3-J2, and C33-A cells were grown in Dulbecco’s Minimal Essential Medium supplemented with 1% penicillin/streptomycin and 10% fetal bovine serum. Transfections were performed using Lipofectamine 2000 according to manufacturer instructions. DNA amounts used in transfections are specified in the figure legends.

### Transcription assay

C33-A cells were transfected with an E2-responsive luciferase reporter and either WT or mutant pCI-V5-E2. Approximately 48 h post-transfection, cells were lysed and luciferase assay was performed according to manufacturer instructions (Promega). Firefly luciferase luminescence was measured on a PHERAstar plate reader and values were calculated as fold change over cells transfected with the reporter alone to account for background luminescence. Assays were performed as three independent replicates. Significance was determined by paired *t*-test between the WT and mutant samples.

### Transient DNA replication assay

Transient replication assays were performed in C33-A cells as described previously (27, 50). Reporter vector concentrations were chosen based upon those used in previous studies (27, 51). E1 and E2 vector concentrations were chosen based upon a titration curve to find the optimal ratio (data not shown). A 2:1 E1:E2 ratio was chosen to favor transient replication over E2-mediated transcription (52, 53).

Approximately 72 h post-transfection, cells were lysed and Renilla and firefly luciferase luminescence was measured on a PHERAstar plate reader. Values were calculated as the ratio of firefly to Renilla luciferase and normalized to cells transfected with the reporters alone. Assays were performed three separate times with eight technical replicates each. Paired *t*-tests between samples with and without E1 were performed to determine significance.

### Immunoblotting and immunofluorescence

For immunoblot experiments, C33-A cells were lysed approximately 48 h post-transfection in cell culture lysis buffer (Promega) and boiled in 3× Laemmli buffer. Whole-cell lysates were separated by SDS-PAGE and transferred to 0.45-µM polyvinylidene fluoride membranes (Millipore) by the semi-dry method. Membranes were blocked in 5% milk in phosphate-buffered saline with Tween (PBST) for 1 h at room temperature. Membranes were then incubated with primary antibodies diluted in PBST overnight. E2 was blotted with V5 (Invitrogen #R960-25, 1:4,000), and alpha tubulin was blotted as a loading control (clone DM1A, 1:5,000). Antibodies were removed and membranes were washed 3× in PBST. Horseradish peroxidase-conjugated secondary antibodies were diluted 1:5,000 in PBST and incubated for 2 h at room temperature. Blots were developed with ECL reagent (Amersham) and detected with a ChemiDoc XRS + Imager (Bio-Rad).

Immunofluorescence was performed by plating HEK293TT cells on glass coverslips and transfecting with 3 μg of either WT or C307 mutant pCI-V5-E2 expression vectors. Approximately 48 h post-transfection, media were removed, and cells were washed with PBS prior to being fixed with 4% paraformaldehyde. After fixation, cells were permeabilized with 0.1% Triton-X100 in PBS for 10 minutes. Permeabilization solution was removed and cells were incubated in blocking solution (PBS + 0.1% Triton-X100 + 10% normal goat serum) for approximately 1 h. Primary rabbit anti-V5 antibody (CST D3H8Q) was added at an approximate 1:1,200 dilution and incubated rocking overnight at 4°C. Primary antibody was removed; cells were washed 3× in PBS; and then cells were incubated with AlexaFluor 555 Goat anti-rabbit (#A21428) at a dilution of 1:1,500 for approximately 2 h at room temperature and protected from light. The secondary antibody was removed; cells were washed 3× in PBS; and coverslips were mounted with ProLong Gold with DAPI.

### Chromatin immunoprecipitation

C33-A cells were transfected with mFLori with or without expression vectors for WT or mutant E2. Approximately 48 h post-transfection, cells were formaldehyde fixed and processed according to manufacturer instructions (Active Motif #53009). Immunoprecipitation was performed using antibodies to V5 (Invitrogen #R960-25) or mouse IgG as a control. Eluted chromatin was quantified with real-time PCR using primers flanking either the palindromic (Mus LCR Fwd: CTGGCAATTATCCGCGTACCG and Mus LCR Rev: GTACGACCGTTCCAGAACACC) or alternative (E2BS alt Fwd: CGGTCGCATATAAGTATCAGTGTGC and E2BS alt Rev: AAAGGGAGCCCCCGATTTAG) E2 binding sites in the MmuPV1 LCR. Values were calculated as fold enrichment over the IgG control, and significance was determined using Student’s *t*-test.

### *In vivo* wart formation

*In vivo* experiments were conducted in homozygous Hsd:Athymic Nude-Foxn1^nu^ mice procured from Inotiv and housed in the IU School of Medicine Laboratory Animal Resource Center. Mice were anesthetized using isoflurane, and tails were wounded using autoclaved sandpaper approximately 72 h prior to injection to enrich for proliferative cells and enhance deposition of extracellular matrix (54, 55). Approximately 18 µg of circular viral genome was injected intradermally in the pre-wounded site using an insulin syringe. Mice were monitored weekly to assess disease progression for 5.5 months. At the end of the study, mice were sacrificed; pictures were taken; and tissues were collected for further analysis.

### Statistical analysis

All data presented were gathered from a minimum of three independent assays. All statistical tests were performed as specified using Microsoft Excel.

## Data Availability

The data and constructs generated during this study are available from the corresponding author upon request.
